# Microenterprise Intervention to Reduce Sexual Risk Behaviors and Increase Employment and HIV Preventive Practices Among Economically-Vulnerable African-American Young Adults (EMERGE): A Feasibility Randomized Clinical Trial

**DOI:** 10.1007/s10461-020-02931-0

**Published:** 2020-06-03

**Authors:** Larissa Jennings Mayo-Wilson, Jessica Coleman, Fatmata Timbo, Fred M. Ssewamala, Sebastian Linnemayr, Grace T. Yi, Bee-Ah Kang, Matthew W. Johnson, Gayane Yenokyan, Brian Dodge, Nancy E. Glass

**Affiliations:** 1 Indiana University School of Public Health, Department of Applied Health Science, 1025 E. 7th Street, Bloomington, IN USA; 2 Johns Hopkins University School of Public Health, Department of International Health, 615 N. Wolfe Street, Baltimore, MD USA; 3 The Brown School, Washington University in St. Louis, Goldfarb, One Brookings, Drive, St. Louis, MO USA; 4 RAND Corporation, 1776 Main Street, Santa Monica, CA USA; 5 Johns Hopkins University School of Medicine, Department of Psychiatry and Behavioral Sciences, 5510 Nathan Shock Drive, Baltimore, MD USA; 6 Johns Hopkins Bloomberg School of Public Health, Department of Biostatistics, 615 N. Wolfe Street, Baltimore, MD USA; 7 Johns Hopkins University School of Nursing, 525 N. Wolfe Street, Baltimore, MD USA

**Keywords:** HIV, Sexual risk behaviors, Homeless, Text messages, Young adults, Baltimore, African-American, Economic, Unemployment, Feasibility, Clinical trial

## Abstract

Economic vulnerability, such as homelessness and unemployment, contributes to HIV risk among U.S. racial minorities. Yet, few economic-strengthening interventions have been adapted for HIV prevention in this population. This study assessed the feasibility of conducting a randomized clinical trial of a 20-week microenterprise intervention for economically-vulnerable African-American young adults. *E*ngaging *M*icroenterpris*E* for *R*esource *G*eneration and Health *E*mpowerment (EMERGE) aimed to reduce sexual risk behaviors and increase employment and uptake of HIV preventive behaviors. The experimental group received text messages on job openings plus educational sessions, mentoring, a start-up grant, and business and HIV prevention text messages. The comparison group received text messages on job openings only. Primary feasibility objectives assessed recruitment, randomization, participation, and retention. Secondary objectives examined employment, sexual risk behaviors, and HIV preventive behaviors. Outcome assessments used an in-person pre- and post-intervention interview and a weekly text message survey. Several progression criteria for a definitive trial were met. Thirty-eight participants were randomized to experimental (n = 19) or comparison group (n = 19) of which 95% were retained. The comparison intervention enhanced willingness to be randomized and reduced non-participation. Mean age of participants was 21.0 years; 35% were male; 81% were unemployed. Fifty-eight percent (58%) of experimental participants completed ≥ 70% of intervention activities, and 74% completed ≥ 50% of intervention activities. Participation in intervention activities and outcome assessments was highest in the first half (~ 10 weeks) of the study. Seventy-one percent (71%) of weekly text message surveys received a response through week 14, but responsiveness declined to 37% of participants responding to ≥ 70% of weekly text message surveys at the end of the study. The experimental group reported higher employment (from 32% at baseline to 83% at week 26) and lower unprotected sex (79% to 58%) over time compared to reported changes in employment (37% to 47%) and unprotected sex (63% to 53%) over time in the comparison group. Conducting this feasibility trial was a critical step in the process of designing and testing a behavioral intervention. Development of a fully-powered effectiveness trial should take into account lessons learned regarding intervention duration, screening, and measurement.

*Trial Registration* ClinicalTrials.gov. NCT03766165. Registered 04 December 2018. https://clinicaltrials.gov/ct2/show/NCT03766165

## Introduction

Economic vulnerability, such as homelessness and unemployment, contributes to HIV risk among racial minorities in the United States (U.S.) who are disproportionately infected. According to UNAIDS, the U.S. has a concentrated HIV epidemic that has greatly affected impoverished urban areas [1, 2] where HIV prevalence is alarmingly high at 2.1%, over seven times the national HIV prevalence (0.3%) [1, 3, 4]. HIV prevalence is 2.1 times higher among individuals with income at or below the U.S. poverty threshold as compared to those above [1, 2], and 2.6 times higher among unemployed individuals as compared to employed individuals [1, 2]. Homelessness in the past 1 year is also associated with 1.8 times higher HIV prevalence [1, 2].

Racial minorities, particularly African-Americans, are disproportionately affected by the HIV epidemic. Although representing 12% of the U.S. population [3], African-Americans make up 42% of all U.S. HIV infections [5]. The rate of new HIV infections is 8.3 times higher in African-Americans compared to non-Hispanic whites [5]. In Baltimore, Maryland (MD), the setting for this study, 82% of adult and adolescent HIV diagnoses were in non-Hispanic Blacks (African-American) [4]. In addition, young adults in Baltimore, MD, aged 20–29, made up the largest proportion of HIV diagnoses (29%) compared to any other age group [4] as well as an increasing proportion of the urban homeless and unemployed.

Economic vulnerability is associated with behaviors that contribute to HIV risk, such as condomless sex and sex exchange [6–8]. Low economic resources may also lead to a loss of hope and agency that diminishes motivations to avoid exposure to future HIV infection [6–8]. Yet, despite persistent racial and economic disparities in HIV infection, few economic-strengthening interventions have been adapted for HIV prevention in economically-vulnerable African-American young adults [9]. The absence of published studies conducted within communities of color has hindered efforts to reduce economic drivers of HIV risk in this population.

Microenterprise (or very small-scale businesses) has been shown to improve sexual attitudes [10–12], sexual risk behaviors [13–20], and HIV communication and testing [13, 15]. Prior microenterprise initiatives have combined HIV and microbusiness training, mentoring, and small grants. However, with a few exceptions, most microenterprise interventions have been conducted outside of the U.S. in low-income countries and using face-to-face classes [21]. They have also focused primarily on female sex workers and older adults (aged 25 to 49) and have required all participants to engage in an identical income-generating activity [21]. Less is known about how to effectively design choice-based microenterprise interventions that are tailored to young adults’ interests and abilities. This is particularly true for disconnected youth who are out of school and unemployed. Our prior research found that young adults experiencing homelessness had access to cell phones and had interests in expanding their current entrepreneurial activities [22, 23].

The objective of this study was to assess the feasibility of conducting a trial of a microenterprise intervention, entitled *E*ngaging *M*icroenterpris*E* for *R*esource *G*eneration and Health *E*mpowerment (EMERGE). We designed EMERGE for economically-vulnerable African-American young men and women in a U.S. urban setting. EMERGE included face-to-face sessions that were augmented by text messaging, grants, and mentoring. While text messages have been used in HIV risk reduction programs [24–27], they have not previously been combined with a microenterprise intervention. The primary feasibility objectives were to assess participant recruitment, randomization, participation, and retention, in addition to intervention acceptability. Secondary exploratory objectives were to examine the level of employment, sexual risk behaviors, and HIV preventive practices in study groups over time. This feasibility study will inform whether and how to conduct a larger effectiveness trial for HIV risk reduction in this population [28]. Findings will help to address uncertainties that would arise when planning a larger trial, such as participant willingness to be randomized, time needed to collect data, intervention acceptance, and responsiveness to outcome assessments [29, 30]. This article presents results of feasibility and behavioral data. Qualitative acceptability data have been published elsewhere [31].

## Methods

### Design

This feasibility trial was a two-group parallel design with a 1:1 allocation ratio to experimental or comparison group.

### Study Registration

**Fig. 1:**
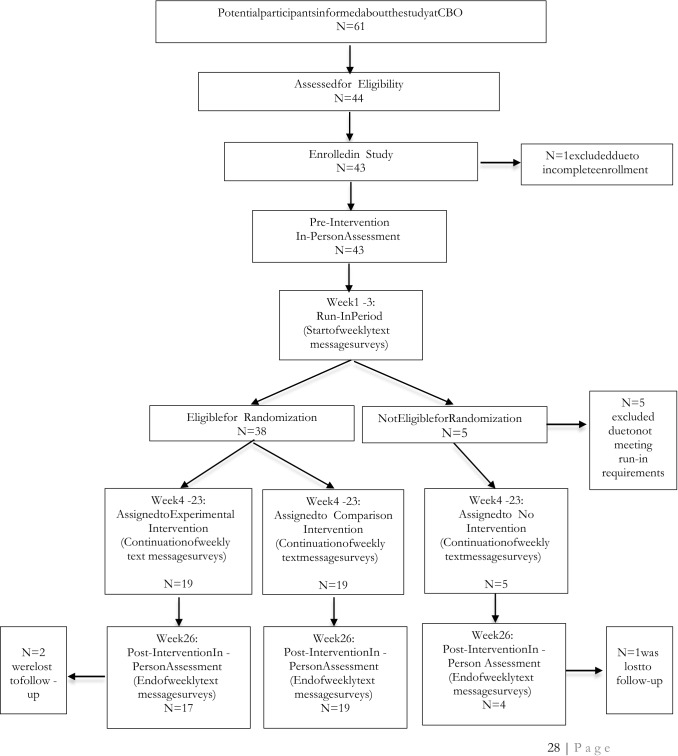
Participant flow diagram

The trial was registered at ClinicalTrials.gov (NCT03766165). It was entitled the EMERGE Project, *E*ngaging *Mi*croenterpris*E* for *R*esource *G*eneration and Health *E*mpowerment (K01MH107310). This manuscript has been prepared according to the Consolidated Standards of Reporting Trials Statement for Social and Psychological Interventions (CONSORT-SPI) [32] and the extension for randomized pilot and feasibility trials [33]. A standard CONSORT diagram is included (Fig. 1). The study methods are based on the EMERGE trial protocol (Version 5; 23 January 2019), approved by the Johns Hopkins University School of Public Health Institutional Review Board (#00008833) and the Indiana University Institutional Review Board (IRB) (#2003950305).

### Setting

The feasibility trial took place in Baltimore, MD in collaboration with two community-based organizations (CBO), AIRS and YO!Baltimore, which provide transitional and emergency housing for young adults who are experiencing homelessness. Participants were recruited from and received the interventions at the CBOs. Data were also collected at the CBOs. The CBOs were chosen based on years working in Baltimore (> 2 years), support to African-American youth experiencing homelessness (> 20 youth per year), and willingness to participate in the study.

### Participants

Details of the study’s participant recruitment process have been published previously in a protocol manuscript [34]. Study eligibility was determined using a screening tool during an in-person enrollment process. The eligibility criteria for participants were: African American, aged 18–24, living in Baltimore, having experienced at least one episode of homelessness in the last 12 months (e.g., defined as reporting any episode in which a person lacked a regular or adequate nighttime residence, such as living in a hotel/motel, vehicle, shelter, or friend’s home and living primarily on their own, apart from a parent or guardian), unemployed or employed fewer than 10 h per week, not enrolled in school, owning a cell phone with text messaging functionality, and reporting at least one episode of unprotected or unsafe sex in the prior 12 months.

Potential participants were recruited on-site at the two participating CBOs. A one-page recruitment flyer was posted in the main building of each CBO. Designated CBO staff informed potential participants of the study team’s scheduled visit days. On these visit days, the PI and/or a trained research assistant introduced the study to a group of potential participants. On the same day, the PI or a trained research assistant then accompanied each potential participant to a private room to complete the screening tool, administer written informed consent, register the participant’s cell phone to the study’s online text messaging service (TextIt.in), and conduct the pre-intervention assessment.

### Timeline

Data were collected for 26 weeks. Participants underwent an in-person, pre-intervention assessment at the time of enrollment and were randomized in week 4 if they successfully completed a three-week run-in period (week 1 to week 3). Both groups received the assigned interventions concurrently for 20 weeks (week 4 to week 23). An in-person post-intervention assessment was conducted in week 26. Participants additionally completed a weekly text message survey (week 1 to week 26) on Fridays for a sub-set of outcomes. Recruitment and pre-intervention assessments began in December 2018 and were completed in February 2019. The run-in period, weekly text message survey, and interventions were delivered from February to July 2019. The post-intervention assessment was conducted from July to August 2019.

### Run-In Period

Following the recruitment period, participants began a three-week run-in period prior to randomization. The run-in period was used to minimize dropouts after randomization by identifying participants who were likely to take-up the intervention and to complete outcome assessments [35–40]. The two run-in requirements were: (1) to respond to one or more of the first three weekly text message surveys and (2) to describe the type of microenterprise one would like to start (e.g., during the enrollment interview or by email/hand-written document). A prior third run-in requirement of attending a group session was omitted to reduce research burden and minimize delays in the time of enrollment until the start of the intervention. Participants who did not successfully complete the run-in requirements were eligible to choose either to withdraw from the study or to remain in the study and only complete the study’s assessments.

### Interventions

Participants assigned to the experimental intervention received the following: (1) one text message each week on job openings in Baltimore, appropriate for young adults at or slightly above or below high school diploma or equivalent training. Text messages on job announcements were sent every Monday at 6PM; (2) one 2-h educational session each week on business start-up and HIV prevention on Wednesdays at 10:30AM or 3:00PM in a classroom at the CBO resource center with a PowerPoint presentation, handouts, group discussions, guest speakers, small-group activities, and completion of the session and participant checklists. The sessions were led by three female facilitators. A text message reminder to attend the educational session was sent 2 h prior to the session; (3) one mentor during the intervention period, who was aged 25 and older, lived in the Baltimore greater metropolitan area, owned a small business in Baltimore, and spoke English. Mentors included men and women who were matched according to the microbusiness interests of the participants; (4) one microbusiness start-up grant (repayment not required) in the amount of $1100.00 paid by check in a series of small payments over the study period and used for purchasing microbusiness supplies, marketing, communication, and travel. Participants were required to attend educational sessions, complete weekly text message surveys, and provide evidence of prior microbusiness purchases (e.g., receipts, goods, photos, etc.) to receive the next small payment; and (5) three text messages each week on microenterprise and HIV prevention, delivered every Tuesday, Wednesday, and Thursday at 6PM to reiterate key messages from the educational sessions. In a larger effectiveness trial, the experimental intervention was hypothesized to work to reduce sexual risk behaviors and increase employment and uptake of HIV preventive behaviors by building skills and providing motivational messaging and financial support.

The proposed experimental intervention was delivered as planned with some minor modifications. All text messages on job openings (20 texts) and microenterprise and HIV prevention (57 texts) were delivered as planned. However, no microenterprise and HIV prevention text messages were delivered during the 1st week of the intervention so as to allow participants to focus on orientation. All 20 educational sessions were delivered as planned. However, the mean time of the weekly sessions was modified to 2 h rather than 3 h based on feedback from the CBO staff and participants after the first two sessions were completed. Microbusiness grants in the amount of $1100 were available for all participants as planned. The final number and pace of payments was determined while the study was underway and included three payments: $500, $300, and $300 in sessions 7, 14, and 17, respectively. Participants were offered a mentor as planned, although the majority of mentors had limited capacity to provide a paid apprenticeship. A total of eight microbusiness individual mentors were recruited in addition to three microbusiness group mentors (e.g., guest speakers during the educational sessions) and two HIV prevention care specialists (e.g., also guest speakers). Mentor matches were made after session 4, as planned, once participants finalized a microbusiness endeavor. There was approximately a two- to three-week lag from the time of the mentor match to the time of initial contact.

**Fig. 2:**
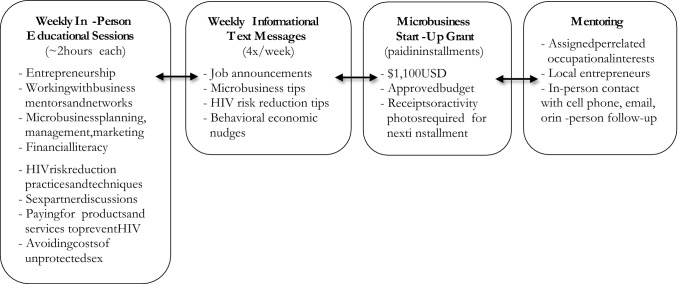
Summary of components of the 20-Week EMERGE experimental intervention

Participants assigned to the comparison intervention received one text message each week on job openings, identical in content and timing (every Monday) to the job openings sent to participants assigned to the experimental intervention. The comparison intervention was implemented as planned with no modifications. Figure 2 highlights components of the experimental intervention. Additional details regarding both interventions, including rationale, structure, and processes are published in the aforementioned protocol manuscript [34].

### Outcomes

To measure feasibility we examined rates of recruitment, randomization, and retention. We also assessed participation in intervention activities and outcome assessments. Specifically, the two primary outcomes of feasibility were: (1) proportion of participants in both groups who responded to 70% or more of the weekly text message surveys measured at week 26; and (2) proportion of experimental intervention participants who completed 70% or more of intervention activities measured at week 23. Core intervention activities included four measures: session attendance, receipt of one or more informational text messages, receipt of one or more mentor contacts, and spending of one or more grant payments.

The secondary feasibility outcomes were: (3) proportion of all participants who received one or more informational text messages measured weekly at weeks 4 to 23; (4) proportion of all participants who responded to the text message survey measured weekly at weeks 1 to 26; (5) proportion of experimental intervention participants who attended an educational session measured weekly at weeks 4 to 23. Participants who missed a session but completed a missed session review and make-up materials during the following session received credit for the session; (6) proportion of experimental intervention participants who received one or more mentor contacts measured weekly at weeks 4 to 23 for any discussion with a mentor or guest speaker in-person or by email, text, online, or phone call; (7) proportion of experimental intervention participants who spent one or more grant payments measured weekly at weeks 4 to 23.

We also explored the completion and level of sexual risk behaviors, HIV preventive behaviors, and employment. Specifically, the secondary behavioral outcomes were: (8) proportion of participants in each group who reported one or more unprotected sex acts in the last week measured weekly at weeks 1 to 26; (9) proportion of participants in each group who reported one or more unprotected sex acts in the last month measured at week 1 and 26; (10) proportion of participants in each group who reported one or more safer sex acts in the last week measured weekly at weeks 1 to 26; (11) proportion of participants in each group who reported one or more safer sex acts in the last month measured at week 1 and 26; (12) proportion of participants in each group who reported one or more HIV preventive care-seeking or information-seeking acts in the last week measured weekly at weeks 1 to 26; (13) proportion of participants in each group who reported one or more HIV preventive care-seeking or information-seeking acts in the last month measured at week 1 and 26; (14) proportion of participants in each group who reported one or more paid hours of work in the last week measured weekly at weeks 1 to 26; (15) proportion of participants in each group who reported one or more paid hours of work in the last month measured at week 1 and 26. The list of eligible behaviors for each behavioral outcome is included in the footnotes of Tables 4 and 5.

Outcome data were collected using responses from the online text messaging service (#1, 3, 4), weekly participant checklists completed at the end of each educational session (#2, 5, 6, 7), weekly text message surveys (#8, 10, 12, 14), and in-person pre-intervention and post-intervention assessments (#9, 11, 13, 15). The pre-intervention assessment was also used to obtain participant demographic data relating to age, gender, sexual orientation, employment status, parental status, prior night’s residence, income insecurity (e.g., having enough money to buy food, housing, and/or transportation in the last 30 days), access to a banking account, and cell phone behaviors. No changes to the feasibility trial’s assessments or measurements were made after the feasibility trial commenced.

### Progression Criteria to Definitive Trial

The decision of whether and how to proceed to a full-scale trial is based on the feasibility data and overall study experience. Progression criteria to a definitive trial that were considered were: (i) recruitment of ≥ 80% of target sample size; (ii) response of ≥ 70% of participants in experimental and comparison groups to ≥ 70% of weekly text message surveys; (iii) completion of ≥ 70% of experimental participants of ≥ 70% experimental intervention activities, such as text message receipt, session attendance, grant spending, and mentor contact; and (iv) acceptability of the comparison and experimental interventions. Acceptability is analyzed and reported in a second manuscript of qualitative findings [31]. Progression criteria also considered retention and the practicality of potential modifications required for an effectiveness trial.

### Sample Size

A power calculation to estimate sample size was not appropriate for a feasibility trial because the aim of the trial was not to establish effectiveness [41]. Instead we determined that a minimum sample of 30 participants (15 in each group) would generate sufficient data to assess trial feasibility and to assess the acceptability of the assigned interventions. This was determined with reference to recommendations for feasibility studies, which recommended sample sizes of between 24 and 50 [41–43] and as used in other published feasibility randomized clinical trials [44–47]. The minimum target sample size of 30 was inflated to 40 to allow for drop-out during the run-in period (15%) or drop-out following randomization (10%), which was estimated to be a total of 25% based on similar studies [35–37, 48].

### Allocation

The PI and a trained research assistant enrolled participants prior to the run-in period and prior to the assignment to comparison or experimental intervention. A biostatistician who was not involved in recruitment, intervention implementation, or outcome assessment used a computer to generate the random allocation sequence and assigned all participants, at the same time, to comparison or experimental intervention. Participants were randomized in a 1:1 ratio, stratified by CBO to allow equal numbers of CBO participants in each study group.

### Masking

A fully masked design was not possible because participants knew to which intervention group they had been assigned, and the study team members administering the microenterprise intervention knew participants’ assignments. However, as both interventions were economic-strengthening activities, the similarities of the interventions were intended to reduce possible biases in the expectations of benefits by participants. Both interventions were described as novel activities aiming to improve employment for young adults in Baltimore.

In addition, the PI provided a masked dataset to two research analysts so that statistical analyses could be performed without knowing group assignments.

### Statistical Analysis

Descriptive statistics were reported for the primary and secondary feasibility outcomes. Frequencies and proportions were used to summarize categorical data for the specified time points by study group and total. Study enrollment, run-in eligibility, randomization, and retention are described using a standard CONSORT diagram (Fig. 1). To improve the interpretation of trial results, ancillary analyses were used to examine trends in responsiveness to the text message survey and in levels of intervention participation. We partitioned primary outcome #1 into three time periods: up to the first quarter of the study period (weeks 1 to 7), up to the second quarter (weeks 1 to 14), and up to the third quarter (weeks 1 to 21). For primary outcome #2, we also calculated the mean percent of intervention activities engaged in and the proportion of experimental participants who completed ≥ 70% and ≥ 50% of all intervention activities, which included the four core activities plus one other microbusiness activity (e.g., developing a business plan, obtaining licensing, sharing products, or preparing a business social media page) and one other intervention text-messaging activity (e.g., sharing texts with peers or business advertising and/or reporting using texts). Inclusion of additional intervention activities enabled the study team to account for participant efforts not previously reflected in the primary outcome. Participants who completed their in-person post-intervention assessment between weeks 26 to 30 were treated as non-missing. Participants who responded to the weekly text message survey by Monday (approximately 72 h after the survey was sent) were treated as non-missing.

Descriptive statistics were also reported for secondary behavioral outcomes, using frequencies and proportions by study group for the specified time points. Given the lower response rates (< 50%) to the weekly text message surveys during the second half of the study period (weeks 15 to 26), prespecified statistical analyses across exposure periods using a random effects generalized linear model were not possible. Therefore, as prespecified if weekly text message survey data were insufficient over time, we compared pre- and post-intervention secondary behavioral outcomes using two tests of proportions with an interaction term of group and time. The coefficient of the interaction term equaled the difference in the change over time in the experimental group minus the change over time in the comparison group. As this was a feasibility trial testing the methods to be used on a larger scale, comparative analyses were performed for the purpose of reporting the level and change difference in behavioral outcomes and not for the purpose of significance testing [28, 41]. We completed analyses for the intention-to-treat sample (defined as every participant randomized) and the per protocol sample (defined as only participants completing 70% or more of core intervention activities). Missing data in the in-person post-intervention assessment for two participants was handled by using the last measure carried forward from the weekly text message survey. Other methods that would account for uncertainty due to missingness (e.g., multiple imputation) were not used because the sample size was small.

## Results

### Feasibility Outcomes

#### Recruitment

The study recruited 100% of the target sample size. Over the 6 weeks of recruitment (December 2018 to February 2019), we recruited 44 participants from two CBOs, approximately 22 participants per CBO. We were unable to obtain complete enrollment for one participant given his use of a cell phone number previously registered by another participant. Therefore, enrollment was completed for 43 participants. We informed approximately 61 participants at the CBO center in an open group format of the intervention of which 17 (28%) self-identified themselves as ineligible after hearing the study’s eligibility criteria. These non-participants did not undergo screening. Self-reported ineligibility was anecdotally due to having employment > 10 h week, being aged 25 or older, intending to move out of town in the coming weeks, or not having a cell phone. No screened participants declined participation.

#### Baseline Data

Pre-intervention assessments were conducted for 43 enrolled participants (100%). The mean age was 21.0 years (Table 1). Thirty-five percent (35%) of participants were male. Most participants (70%) had high school diploma or equivalent as their highest level of education. Twenty-eight percent (28%) had only completed up to grades 8 to 11. Two percent (2%) of participants had completed up to 2 years of college. Unemployment and income insecurity were high (81% and 84%, respectively). Housing status varied with 19% of participants having spent the previous night in an emergency shelter compared to 2% with a stranger; 42% in transitional housing; and 33% at the home of a friend, relative, or intimate partner. Five percent (5%) had their own apartment. All participants had access to a cell phone as required for study enrollment. Eighty-four percent (84%) had a password-enabled cell phone. Forty-percent (40%) stated that others could access their cell phone.

**Table 1:** Baseline demographic and socio-economic characteristics of 43 African-American economically-vulnerable young adults allocated to experimental, comparison, and non-randomization groups in the EMERGE feasibility randomized clinical trial

Characteristic	Study group			Total
	Experimental	Comparison	Non-randomization	
Number of participants	19	19	5	43
Male participants	6	7	2	15
Female participants	13	12	3	28
Male	32%	37%	40%	35%
Mean age in years (min, max)	21.3 (18, 24)	20.9 (18, 24)	20.4 (18, 24)	21.0 (18, 24)
Highest level of education				
Grades 8 to 11	32%	16%	60%	28%
High school diploma	63%	84%	40%	70%
2-year college	5%	0	0	2%
4-year college	0	0	0	0
Unemployed	84%	84%	60%	81%
Previous night’s residence				
Emergency shelter (CBO)	32%	5%	20%	19%
Transitional housing (CBO)	37%	42%	60%	42%
With friend, relative, partner	26%	42%	20%	33%
With stranger	0	5%	0	2%
Street/public space	0	0	0	0
Private apartment	5%	5%	0	5%
Currently a parent	16%	11%	60%	19%
Unmarried	100%	95%	100%	98%
Has banking account	47%	53%	0	44%
Income insecurity in last 30 days	90%	74%	100%	84%
Sexual orientation (males)^a^				
Sex with men only	17%	29%	0	20%
Sex with women only	83%	71%	100%	80%
Sex with men and women	0	0	0	0
Sexual orientation (females)^b^				
Sex with men only	92%	83%	100%	89%
Sex with women only	8%	0	0	4%
Sex with men and women	0	17%	0	7%
Has cell phone with enabled password	84%	79%	100%	84%
Others have access to cell phone	37%	37%	60%	40%

^a^Among enrolled men

^b^Among enrolled women

#### Randomization

Figure 1 provides a participant flow diagram. Thirty-eight enrolled participants (88%) completed the run-in requirements and were randomized 1:1 to comparison (n = 19, 50%) or experimental intervention (n = 19, 50%). Five enrolled participants (12%) were ineligible for randomization because they did not respond to one or more of the first three text message surveys as specified by the run-in requirements.

#### Weekly Text Message Survey Participation

Text message survey participation was relatively high in the first half of the study, but declined thereafter (Table 2). Seventy-nine percent (79%, n = 30) of participants in both groups responded to ≥ 70% of weekly text message surveys through week 7, compared to 55% (n = 21) and 45% (n = 17) through weeks 14 and 21, respectively. Through weeks 7 and 14, the proportion of all text message surveys that received a response was 81% and 71%, respectively, among all randomized participants compared to 63% and 59% through weeks 21 and 26, respectively. At the end of the 26-week period, 37% (n = 14) of participants in both groups responded to ≥ 70% of weekly text message surveys. The experimental group had higher response rates in the early weeks of the study. The comparison group had higher response rates in the middle and ending weeks of the study (Table 2).

**Table 2:** Primary feasibility outcomes in N = 38 African-American economically-vulnerable young adults allocated to experimental or comparison group in the EMERGE feasibility randomized clinical trial

Outcome	# Participants analyzed	Week of measurement	# Weeks included	Pre-specified analyses	Ancillary analyses	Study group		Total
						Experimental	Comparison	
Number of participants						19	19	38
Proportion who responded to ≥ 70% of weekly text message surveys	38	7	7		✓	.842	.737	.789
	38	14	14		✓	.474	.632	.553
	38	21	21		✓	.316	.579	.447
	38	26	26	✓		.263	.474	.368
Proportion of text message surveys receiving a response	38	7	7		✓	.805	.812	.808
	38	14	14		✓	.677	.748	.712
	38	21	21		✓	.566	.697	.632
	38	26	26		✓	.520	.660	.590
Proportion who completed ≥ 70% of core intervention activities^a^	19	23	20	✓		.579^c^	–	–
Proportion of who completed ≥ 70% of all intervention activities^b^	19	23	20		✓	.579	–	–
Proportion who completed ≥ 50% of all intervention activities^b^	19	23	20		✓	.737	–	–
Mean percent of all intervention activities engaged in^b^	19^d^	23	20		✓	.693	–	–
	11^c^	23	20		✓	.909	–	–

^a^Core intervention activities included text receipt, session attendance, mentor contact, and grant spending

^b^All intervention activities included core interventions plus two additional interventions (any micro-business activity and any intervention text-messaging activity)

^c^Per protocol sample

^d^Intention-to-treat sample

#### Intervention Participation

Intervention participation was also high in the first half of the study, but declined thereafter. The majority of participants (> 50%) engaged in text-messaging, educational sessions, grant spending, and mentor contact up to weeks 8 to 14 of the intervention (Table 3). Most experimental participants (65% to 71%) attended educational sessions during the first half of the intervention (weeks 1 to 9). During the second half (weeks 10 to 20), session attendance was lower and varied from 47 to 18%. Mentor contacts among experimental participants commenced in week 6 at 58% and varied from 58 to 11% over time. Grant spending followed similar trends with 58% of participants spending grant monies up to week 13, and declining to 26% in the remaining weeks. Among comparison and experimental participants, 100% received one or more informational text messages during the intervention period, such as job announcements only or job announcements and HIV and microbusiness tips.

**Table 3:** Secondary feasibility outcomes for 20-week intervention period in N = 38 African-American economically-vulnerable young adults allocated to experimental or comparison group in the EMERGE feasibility randomized clinical trial

Outcome										
Intervention week #	1	2	3	4	5	6	7	8	9	10
Text receipt										
Proportion of all participants who received ≥ 1 informational texts	1.00	1.00	1.00	1.00	1.00	1.00	1.00	1.00	1.00	1.00
Survey response										
Proportion of all participants who responded to the text message survey	.82	.82	.76	.74	.61	.58	.66	.66	.61	.61
Session attendance										
Proportion of experimental participants who attended a session	.71	.71	.71	.59	.65	.59	.65	.59	.65	.47
Mentor contact										
Proportion of experimental participants who received ≥ 1 mentor contacts	–	–	–	–	–	.58	.58	.42	.21	.37
Grant spending										
Proportion of experimental participants who spent ≥ 1 grant payments	–	–	–	–	–	–	.58	.58	.58	.58

Outcome										
Intervention week #	11	12	13	14	15	16	17	18	19	20
Text receipt										
Proportion of all participants who received ≥ 1 informational texts	1.00	1.00	1.00	1.00	1.00	1.00	1.00	1.00	1.00	.97
Survey response										
Proportion of all participants who responded to the text message survey	.61	.61	.61	.47	.42	.42	.39	.37	.39	.45
Session attendance										
Proportion of experimental participants who attended a session	.47	.41	.35	.29	.29	.18	.18	.24	.24	.35
Mentor contact										
Proportion of experimental participants who received ≥ 1 mentor contacts	.16	.21	.11	.11	.26	.21	.16	.11	.11	.11
Grant spending										
Proportion of experimental participants who spent ≥ 1 grant payments	.58	.58	.58	.32	.32	.32	.26	.26	.26	.26

In summary, 58% (n = 11) of experimental participants completed ≥ 70% of the core intervention activities (Table 2). This subset defined the per protocol experimental sample. Seventy-four percent (74%, n = 14) of experimental participants completed half of all intervention activities. The mean percent of all activities completed was 69% and 91% in the intention-to-treat and per protocol samples, respectively. Intervention activities with the greatest participation were text messaging (100%), microbusiness activity (74%), and session attendance (74%).

Twenty-six percent (26%, n = 5) of experimental participants never attended an educational session. Reported reasons for session non-attendance included new or changing employment, other personal obligations, competing activities at the CBO, lack of transportation, or “not feeling like it”. In addition, two experimental participants (11%) were later disallowed to visit the CBO due to disputes with other CBO staff and youth and therefore unable to attend the educational sessions, although they continued to receive the intervention’s text messages and assessments. Grant spending was limited by eligibility (e.g., sending in receipts and few educational session absences). Mentor contact was limited by delays in the identification of mentors with comparable occupational interests and scheduling conflicts between mentors and mentees. Reported reasons for decreases in overall intervention participation in the second half of the study were feeling overwhelmed with other personal obligations, feeling this was not a good time to start a microbusiness, and being ineligible to receive future grant payments. At the end of the study, 82% (n = 14) of experimental participants and 74% (n = 14) of all comparison participants reported one or more positive experiences with EMERGE. Additional data on acceptability have been reported elsewhere [31].

#### Retention

Post-intervention assessments were completed by 93% (n = 40) of enrolled participants (Fig. 1). The remaining three participants (7%) were lost-to-follow up. Retention among randomized participants was 95% (n = 36) compared to 80% (n = 4) among non-randomized participants who did not meet the run-in requirements. Retention within randomized participants was 89% (n = 17) of experimental participants and 100% (n = 19) of comparison participants. Total loss-to-follow-up was 19% (n = 8), accounting for 12% (n = 5) during the run-in period and 7% (n = 3) post-randomization. Reasons for post-randomization lost to follow-up were that one participant in the non-randomization group did not respond to the any of the study’s assessment invitations. A second experimental participant was reported to have moved out of town, had no email address, and had a disconnected cell phone. A third experimental participant was no longer allowed on the premises of the CBO and did not attend two scheduled off-site assessments. No participants reported an intervention-related adverse event or a severe adverse event. No participants were withdrawn from the study.

### Behavioral Outcomes

Using the intention-to-treat sample, the experimental group reported higher employment over time in pre- versus post-intervention assessments (32% to 83%) compared to reported changes in employment over time in the comparison group (37% to 47%) (Table 4). The experimental group also reported a larger decrease in unprotected sex over time (79% to 58%) compared to reported changes in unprotected sex over time in the comparison group (63% to 53%). HIV preventive behaviors were high in both groups at pre-intervention and remained high at post-intervention in the experimental group (95% to 100%) while declining in the comparison group (95% to 74%). Both groups also reported decreases over time in safer sex from 79 to 68% in the comparison group and 84% to 53% in the experimental group. However, the experimental group reported higher sexual abstinence over time (16% to 47%) compared to reported changes in sexual abstinence over time in the comparison group (21% to 32%). Levels of secondary behavioral outcomes were comparable in the per-protocol sample (Table 5).

**Table 4:** Secondary behavioral outcomes at pre- and post-intervention using two tests of proportions in intention-to-treat sample (N = 38) of African-American economically-vulnerable young adults allocated to experimental or comparison group in the EMERGE feasibility randomized clinical trial

Behavioral outcome	Intention-to-treat analysis (n = 38)						
	Experimental			Comparison			Change difference
	Pre-	Post-	Δ_E_	Pre-	Post-	Δ_C_	Δ_E_ – Δ_C_
Week #	1	26		1	26		
# of participants	19	19		19	19		19
Unprotected or unsafe sex							
Proportion who reported engaging in one or more unprotected^a^ or unsafe^b^ sex acts in the last month	0.79	0.58	– 0.21	0.63	0.53	– 0.11	– 0.10
Unprotected sex only							
Proportion who reported engaging in one or more unprotected^a^ sex acts in the last month	0.53	0.37	– 0.16	0.37	0.32	– 0.05	– 0.11
Unsafe sex only							
Proportion who reported engaging in one or more unsafe^b^ sex acts in the last month	0.68	0.53	– 0.16	0.58	0.47	– 0.11	– 0.05
Safer sex							
Proportion who reported engaging in one or more safer sex acts in the last month^c^	0.84	0.53	– 0.32	0.79	0.68	– 0.11	– 0.21
Sexual abstinence							
Proportion who reported being sexual abstinent in the last month	0.16	0.47	+ 0.31	0.21	0.32	+ 0.11	+ 0.20
HIV preventive behavior							
Proportion who reported engaging in one or more HIV preventive care-seeking or information-seeking acts in the last month^d^	0.95	1.00	+ 0.05	0.95	0.74	– 0.21	+ 0.26
Any employment							
Proportion who reported doing one or more paid hours of work from job or own business in the last month	0.32	0.83	+ 0.51	0.37	0.47	+ 0.10	+ 0.41
Employment from job only							
Proportion who reported earning money through a job	0.16	0.63	+ 0.47	0.16	0.42	+ 0.26	+ 0.21
Employment from own business							
Proportion who reporting earning money through a self-employed business activity	0.21	0.41	+ 0.20	0.21	0.21	0	+ 0.20

^a^Includes condomless sex without HIV medications

^b^Includes sex while high/drunk, sex with unknown HIV status, sex with concurrent partners, and/or sex exchange)

^c^Includes having had oral sex only, sex while sober, sex with lubricant, sex with one person only, and/or sex with PrEP

^d^Includes having obtained HIV test, acquired condoms, taking HIV medications, and/or discussion of HIV prevention with sex partner, counselor, or in a small group

**Table 5:** Secondary behavioral outcomes at pre- and post-intervention using two tests of proportions in per protocol sample (N = 30) of African-American economically-vulnerable young adults allocated to experimental or comparison group in the EMERGE feasibility randomized clinical trial

Behavioral outcome	Per protocol analysis (n = 30)						
	Experimental			Comparison			Change difference
	Pre-	Post-	Δ_E_	Pre-	Post-	Δ_C_	Δ_E_ – Δ_C_
Week #	1	26		1	26		
# of participants	11	11		19	19		19
Unprotected or unsafe sex							
Proportion who reported engaging in one or more unprotected^a^ or unsafe^b^ sex acts in the last month	0.82	0.55	− 0.27	0.63	0.53	− 0.10	− 0.17
Unprotected sex only							
Proportion who reported engaging in one or more unprotected^a^ sex acts in the last month	0.55	0.36	− 0.18	0.37	0.32	− 0.05	− 0.13
Unsafe sex only							
Proportion who reported engaging in one or more unsafe^b^ sex acts in the last month	0.64	0.55	− 0.09	0.58	0.47	− 0.11	+ 0.02
Safer sex							
Proportion who reported engaging in one or more safer sex acts in the last month^c^	0.82	0.45	− 0.36	0.79	0.68	− 0.11	− 0.25
Sexual abstinence							
Proportion who reported being sexual abstinent in the last month	0.18	0.55	+ 0.36	0.21	0.32	+ 0.11	+ 0.25
HIV preventive behavior							
Proportion who reported engaging in one or more HIV preventive care-seeking or information-seeking acts in the last month^d^	0.91	1.00	+ 0.09	0.95	0.74	− 0.21	+ 0.30
Any employment							
Proportion who reported doing one or more paid hours of work from job or own business in the last month	0.36	0.91	+ 0.55	0.37	0.47	+ 0.10	+ 0.45
Employment from job only							
Proportion who reported earning money through a job	0.18	0.73	+ 0.55	0.16	0.42	+ 0.26	+ 0.29
Employment from own business							
Proportion who reporting earning money through a self-employed business activity	0.27	0.55	+ 0.27	0.21	0.21	0	+ 0.27

^a^Includes condomless sex without HIV medications

^b^Includes sex while high/drunk, sex with unknown HIV status, sex with concurrent partners, and/or sex exchange)

^c^Includes having had oral sex only, sex while sober, sex with lubricant, sex with one person only, and/or sex with PrEP

^d^Includes having obtained HIV test, acquired condoms, taking HIV medications, and/or discussion of HIV prevention with sex partner, counselor, or in a small group

## Discussion

### Principal Findings

This study helped to identify a number of unknown parameters required for the design of a future definitive study. First, we found that the recruitment pace and time needed to collect data were sufficient. We were also able to deliver the full 20-week intervention of educational sessions, grants, mentors, and text messages. Second, we found that participants were willing to be randomized, and the comparison intervention was successful in reducing non-participation. This may have resulted because both interventions were described as novel activities aiming to improve employment for young adults in Baltimore. We also found that some participants were disappointed by assignment to the comparison intervention, which they perceived to have less value. However, they also valued the potential perceived benefits of receiving the comparison group’s job announcements. In fact, the comparison group had higher retention and survey participation rates at the end of the study than the experimental group. This may have occurred because while experimental participants received financial incentives for multiple activities, comparison participants only received incentives for the study’s weekly survey. It is possible that having fewer intervention activities to do over time resulted in greater focus on one activity by the comparison group.

We also found that experimental participation rates were relatively high until weeks 8 to 14 of the intervention, after which they declined. Therefore, a shorter intervention may be preferable for this population. Participants experienced changing cell phone numbers and changing employment and housing situations. Limited time management skills, competing personal obligations, and ongoing interpersonal disputes with CBO peers and cohabiting partners may also have been overwhelming in combination with intervention activities and limited participation over time. Having an equal number of sessions in a shorter time period could enable participants to take advantage of the intervention prior to the onset of further uncertainties or instability. Response rates to the weekly text message surveys were also moderate to high until about 12 to 14 weeks after the start of the interventions. This suggests that shortening the duration of weekly text message assessments in a future trial could complement a shorter intervention period. Having fewer text message questions may also improve long-term responsiveness. Other efforts to maintain intervention participation could include providing payment for travel to sessions, having online sessions, including time management skills training, and assessing readiness to start a microbusiness. Having mentors attend all sessions, rather than a select few, may also enhance participation.

The trial also provided important information concerning retention. The study achieved high retention which may have resulted from the weekly contact to all participants by text message, marketing flyers and emails, and the provision of snacks and payment for the final study assessment. In addition, loss to follow-up among participants who met the run-in requirements was low and less than anticipated. This finding suggests that the two run-in requirements were successful in screening out individuals unlikely to be retained in the study. In contrast, the two run-in requirements had mixed results in excluding individuals who were likely to have low participation as five participants did not attend any educational sessions. Low participation in a future trial could be addressed by assessing readiness or adding attendance to one or more sessions to the run-in requirements. Should the latter be implemented, our findings suggests that sample size calculations would need to be adjusted for higher drop-out prior to randomization, such as 25%. This may be important given that individuals with high engagement early-on were more likely to have high engagement at the end of the study.

Finally, while the trial was not powered to examine effectiveness, the exploratory changes in behavioral outcomes are worth noting. Both the comparison and experimental groups reported increased employment and decreased unprotected sex over time. However, a fully-powered definitive trial is needed to assess the statistical significance of the larger relative changes observed in the experimental group. The weekly text surveys with questions on sexual behaviors may have been an unintended active ingredient of the intervention (e.g., cues, reminders) and contributed to potential changes in the comparison group, along with the weekly job announcements. It is possible also that participants assigned to the comparison group exhibited an increase in performance in order to compete with the experimental group, a phenomenon that has been recorded in trials as “performance bias” [49, 50]. In contrast, the addition of educational sessions, motivational texts, grants, and mentoring may be important factors in assessing effectiveness in a future trial among experimental participants. More research is also needed regarding participants’ decisions to reduce sexual encounters versus use of safer sexual practices within existing relationships. We found that decreases in reported safer sex in the experimental group were coupled with an equal increase in reports of sexual abstinence. In addition, the more positive changes in the per protocol participants suggest that efforts to sustain active participation in a definitive trial may have important effects. There is also a need to investigate potential causal pathways by which economic-strengthening interventions affect HIV risk, such as by increasing negotiating power within sexual relationships, by increasing access to safe housing free of domestic and sexual violence, by decreasing reliance on transactional sex, or by increasing financial access to HIV prevention knowledge and services [51].

### Progression to a Definitive Study

Several of the study’s pre-determined progression criteria for a definitive trial were met. The study was implemented as planned and reached its recruitment target. The study also had minimal loss-to-follow-up and high acceptability among experimental and comparison interventions. However, only 58% of experimental participants had high participation at the end of the study. This was less than the 70% target. In contrast, moderate to high participation was achieved by most participants (74%) in the first half of the study (up to week 14). The study achieved its participation target for text message surveys (70% responding to 70% of text message surveys) up to week 8, but not at the end of the study.

Taken together, these findings support progression to a larger trial with some modifications. One modification is to shorten the duration of the intervention and text message assessment activities to approximately 10 weeks. This is considered practical and consistent with the most active period observed in the trial. Ten weeks is also consistent with participant preferences for more frequent sessions, such as two or more a week, over a shorter time period [31]. Given that the most active participants completed 91% of intervention activities, a second modification may be to improve screening to identify the most active participants. Modifications to screening could include requiring attendance to one or more orientation sessions prior to randomization, assessing intervention readiness, or requiring submission of a reference letter. Including more frequent rewards and mentor support may also be beneficial.

## Limitations

The study’s limitations should be considered. All measures were self-reported and may be subject to reporting biases. Some participants reported technological difficulties in receiving the weekly text message survey due to frozen screens or stalled prompts. These issues may have contributed to lower response rates. A future trial may benefit from giving participants an alternate mode of weekly assessment such as email, phone call, or web should a problem arise with text messages. Another limitation was the relatively small sample size, which was appropriate for a feasibility study, but which did not allow for hypothesis testing [28, 41]. A larger trial with adequate statistical power is needed to examine effectiveness. Having all female session leaders may also have limited the study’s engagement of male participants. To counter this, male mentors and guest speakers were included. Finally, some measurements related to preventive and safer sex behaviors had baseline prevalence of   > 80% which may have led to a ceiling effect given the number of eligible behaviors defined by the study’s protocol [34]. Future assessments may benefit from examining effectiveness using disaggregated outcomes. The study’s strengths were the multiple lessons learned for a future trial relating to recruitment, participation, and retention. Both interventions were conducted in partnership with CBOs, tailored to the needs of participants, and well received. The use of mixed methods of assessment additionally enhanced our evaluation.

## Conclusions

Conducting this feasibility trial was a critical step in the process of designing and testing a behavioral intervention. The trial demonstrated feasibility of the experimental and comparison interventions with promising changes in employment and HIV-related outcomes. Design of an effectiveness trial should take into account the study’s lessons learned with regard to intervention duration, screening, and measurement as these are likely to be important to the success of a larger study. Given that economic vulnerability is associated with HIV among African-American young adults, more research is needed on the role of integrated microenterprise interventions.

## Data Availability

The de-identified dataset used to analyze results for this study and the final study protocol are available in an online repository.

## Abbreviations

AIRS: AIDS Interfaith Residential Services
EMERGE: *E*ngaging *M*icroenterpris*E* for *R*esearch *G*eneration and Health *E*mpowerment
CBO: Community Based Organization
HIV: Human Immunodeficiency Virus
IRB: Institutional Review Board
ITT: Intent-to-treat
MD: Maryland
PP: Per protocol
USD: United States Dollar
YO!B: Youth opportunities! Baltimore
